# The Enzymatic Activity of APOBE3G Multimers

**DOI:** 10.1038/s41598-018-36372-6

**Published:** 2018-12-18

**Authors:** Yangang Pan, Karen Zagorski, Luda S. Shlyakhtenko, Yuri L. Lyubchenko

**Affiliations:** Department of Pharmaceutical Sciences, College of Pharmacy, University of Nebraska Medical Center, Omaha, Nebraska 68198-6025 USA

## Abstract

APOBEC3G (A3G) belongs to the family of cytosine deaminases that play an important role in the innate immune response. Similar to other, two-domain members of the APOBEC family, A3G is prone to concentration-dependent oligomerization, which is an integral for its function in the cell. It is shown that oligomerization of A3G is related to the packing mechanism into virus particle and, is critical for the so-called roadblock model during reverse transcription of proviral ssDNA. The role of oligomerization for deaminase activity of A3G is widely discussed in the literature; however, its relevance to deaminase activity for different oligomeric forms of A3G remains unclear. Here, using Atomic Force Microscopy, we directly visualized A3G-ssDNA complexes, determined their yield and stoichiometry and in parallel, using PCR assay, measured the deaminase activity of these complexes. Our data demonstrate a direct correlation between the total yield of A3G-ssDNA complexes and their total deaminase activity. Using these data, we calculated the relative deaminase activity for each individual oligomeric state of A3G in the complex. Our results show not only similar deaminase activity for monomer, dimer and tetramer of A3G in the complex, but indicate that larger oligomers of A3G retain their deaminase activity.

## Introduction

Human APOBEC3G (A3G) belongs to the family of proteins, which play an important role in the immune response against HIV and other retroviruses and retrotransposons^1,2^. In the absence of viral infectivity factor (Vif) A3G inhibits HIV cycle by deamination of single-stranded DNA (ssDNA)^2^ or by deamination-independent mechanism^3^, creating roadblock of the viral DNA synthesis^4,5^. A3G has two canonical cytidine deaminase domains: N-terminal domain (NTD), which is catalytically non-active and important for binding with ssDNA and RNA and C-terminal domain (CTD), which is responsible for catalytic activity. Similar to other two-domain APOBEC3 family proteins, full length human APOBEC3G has a propensity toward oligomerization^6–8^. Several studies have demonstrated that A3G exists in solution as monomers, dimers and higher order multimers^6,7,9–11^ depending on its concentration in solution^12^ and the important role of oligomerization for antiviral functions of A3G is reviewed in^7,13–15^. It was shown that A3G multimers in complex with RNA, are recruited for packing into viral particle^10,16^ and later confirmed that multimerization correlates with efficient packing of A3G into HIV^17^. Recently Chaurasiya *et al*.^4^ suggested that A3G oligomers are required for deaminase–independent inhibition of viral cDNA replication. However, the effect of oligomerization on enzymatic activity of A3G is remains controversial. Some studies describe A3G oligomerization as essential for its deaminase activity^8,14^, at the same time authors in^15^ claimed that oligomerization decreases enzymatic function of A3G. Even less is known about the deaminase activity for individual oligomers of A3G. Indeed, Shindo *et al*.^5^ tested deletion mutants of A3G and found that all the deletion mutations lost antiviral activity due to the loss of oligomerization activity. At the same time, the authors in^18^ revealed that monomeric A3G, mutated at cysteine residue (C97) in the N -terminal zing-finger motif of A3G, abolished multimerization, but retained enzymatic activity. In 2010, Chelico *et al*.^13^ showed that mutant of A3G, designed to disrupt dimerization is enzymatically active. Several attempts have been made to determine the deaminase activity of individual oligomeric states of A3G. In 2006 study Chelico *et al*.^19^ determined the deamination activity of selected gel-filtration fractions and found the highest deaminase activity for the dimers fraction. Meanwhile, McDougall *et al*.^14^ concluded that tetramers of native full length A3G are more enzymatically active than dimers.

Here we determined the deaminase activity of individual oligomers of A3G in complex with ssDNA, for which the formation has been shown to be a function of the protein concentration^8,11,12,17^. By altering the concentration of A3G during the reaction with ssDNA, we assembled A3G-ssDNA complexes with different A3G stoichiometry. We applied Atomic Force Microscopy (AFM) to determine the yield and stoichiometry of A3G in the complexes, depending on concentration of A3G in the reaction. In parallel, using PCR deaminase assay^10^ we calculated deaminase activity of individual oligomers of A3G. The data obtained for stoichiometry, yield and deaminase activity of individual oligomers of A3G allowed us to compare their deaminase activity. Our data revealed that monomers, dimers and even tetramers have similar deaminase activity, suggesting that oligomerization does not affect deaminase activity of A3G.

## Results

### The Stoichiometry of A3G in Complex with Hybrid DNA

**Figure 1:**
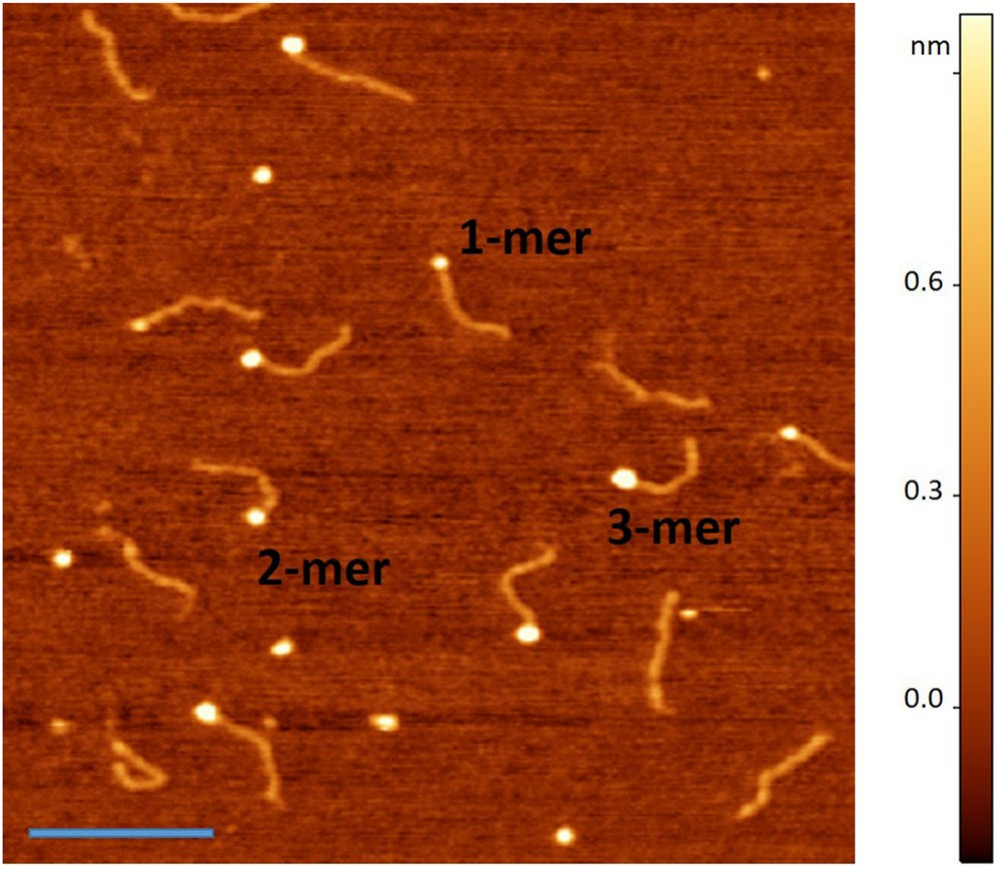
AFM image of A3G in complex with hybrid DNA. AFM image of 16 nM A3G in complex with 4 nM of hybrid DNA. Numbers mark the examples for monomer, dimer, and trimer of A3G in complexes with hybrid DNA. Bar size is 200 nm.

Using AFM imaging and our developed hybrid DNA approach^11,20^, we directly visualized A3G complexes. Figure 1 shows one of the typical AFM images for A3G-DNA complexes at 16 nM A3G and 4 nM DNA. The protein appears as a bright feature (blob) at the end of the dsDNA, and varying brightness and size of the blobs reflect different protein stoichiometry. Using cross-section features, as described in the Material and Methods section, we estimated the volume of the protein, which we converted into molecular weight, using a conversion coefficient 1.3, as determined by Shlyakhtenko *et al*.^21^. Numbers 1, 2, and 3 in Fig. 1 correspond to A3G monomer, dimer and trimer, respectively. For additional A3G concentrations, a set of AFM images was obtained (see Fig. S5).

**Figure 2:**
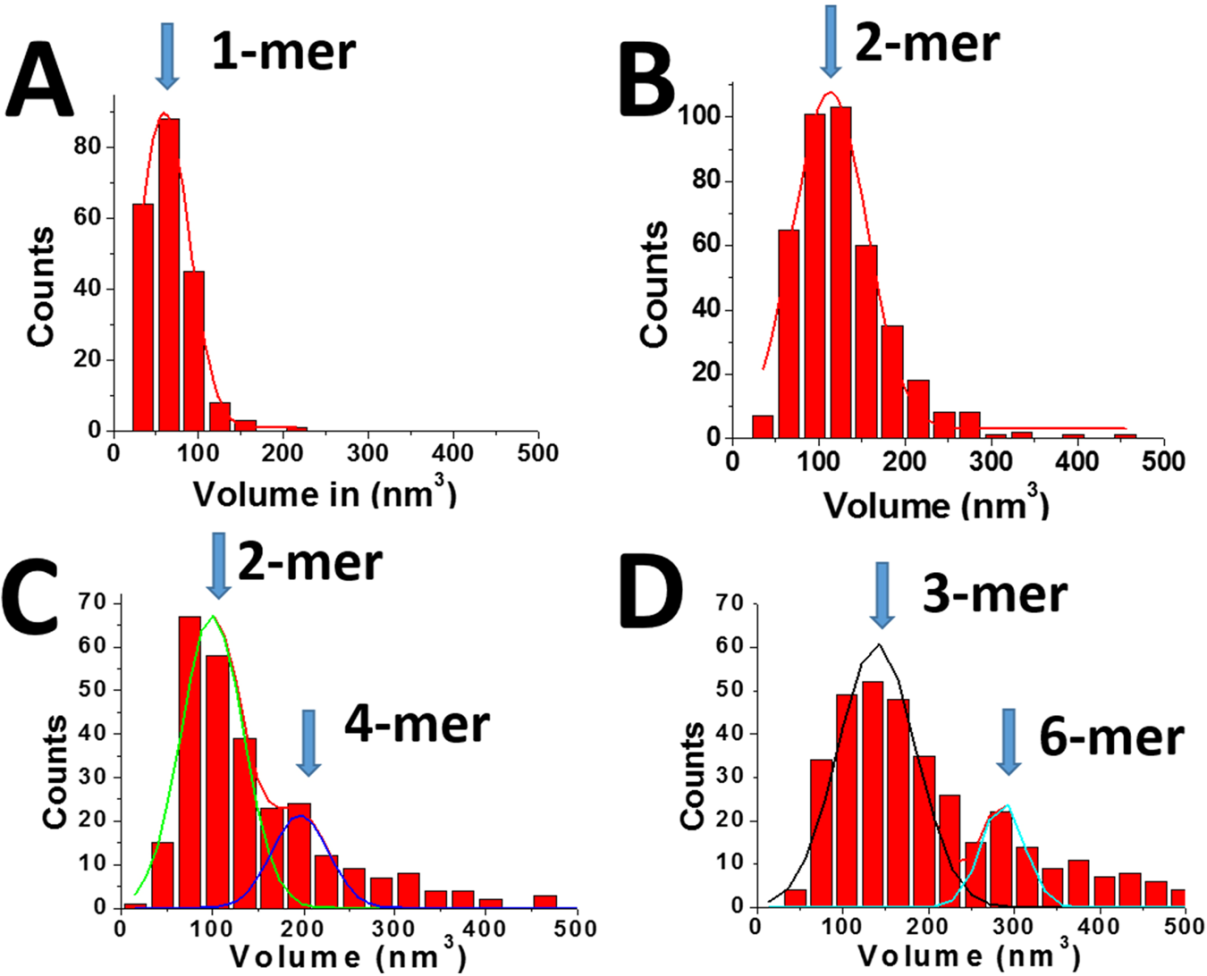
Histograms for A3G volume in complex with hybrid DNA. (**A**) A3G monomer (2 nM). (**B**) A3G dimer (16 nM). (**C**) A3G-78% dimers and 22% tetramers (32 nM). (**D**) A3G: 82% trimers and 18% higher oligomers (80 nM). The ssDNA concentration in all reactions is 4 nM. For each histogram, more than 200 complexes were calculated.

**Figure 3:**
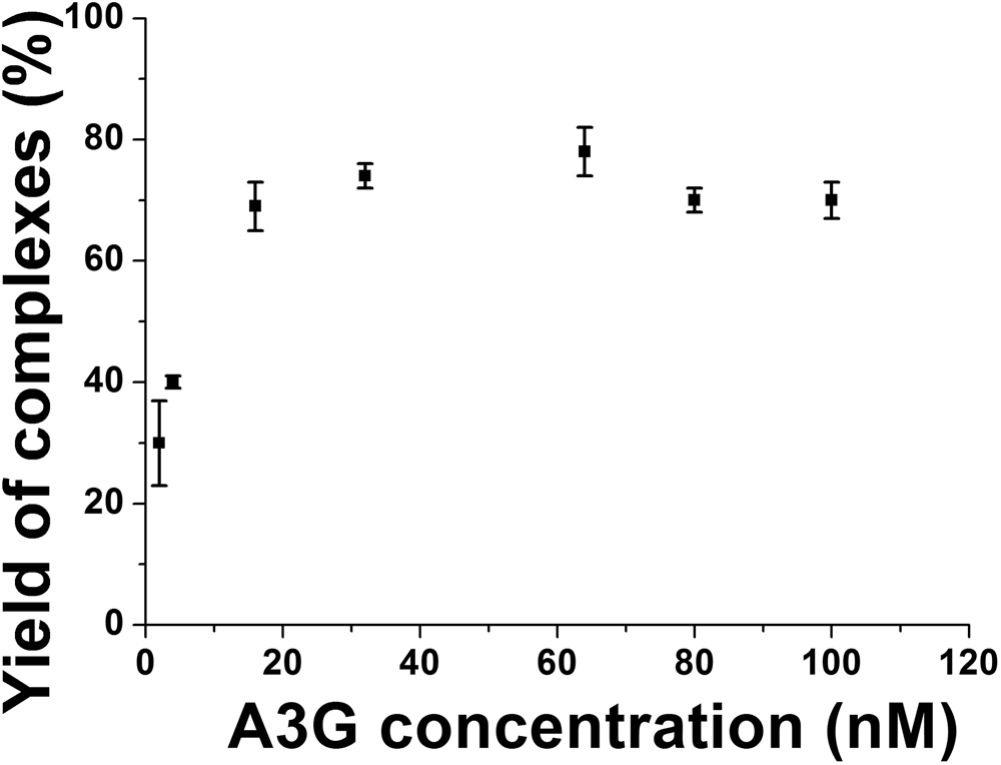
The dependence of the yield of A3G-DNA complexes on A3G concentration. The yield of the complexes at each A3G concentration was calculated from the ratio of the number of A3G-ssDNA complexes to the number of total DNA molecules in each AFM image. The data were obtained from 3 independent experiments and the bar sizes correspond to the variations in the yield values obtained in these independent experiments.

Volumes of A3G protein were estimated, and the data were assembled as histograms. Figure 2 shows histograms for A3G at 2 nM (A), 16 nM (B), 32 nM (C) and 80 nM (D) fitted with Gaussians. In all experiments, the DNA concentration remained constant, at 4 nM. Figure 2A shows the histogram for the lowest A3G concentration (2 nM), with the maximum in the histogram corresponding to volume ~61 nm^3^. After conversion, this value fits to a molecular weight of ~47 kD, which corresponds to A3G monomer. At 16 nM of A3G (Fig. 2B), the volume is about twice larger as the monomer, now totaling ~110 nm^3^, which coincides with A3G dimer. The maximum in the histogram corresponds to the majority of A3G dimers with a small admixture of monomers and trimers as illustrated in AFM image in Fig. 1. At 32 nM of A3G, (Fig. 2C) along with the dimers (volume ~100 nm^3^), a small number of tetramers (volume ~200 nm^3^) appeared. From the ratio of the areas under the Gaussian, we estimated the number of dimers as 78% and tetramers as 22% in this sample. The A3G concentration, 80 nM, as shown in Fig. 2D, produced complexes with 88% trimers of A3G (volume ~150 nm^3^) with admixture of higher order oligomers (12%). The yield of the complexes at each A3G concentration was calculated from the ratio of the number of A3G-ssDNA complexes to the number of total DNA molecules in each AFM image, as described in Methods/Data Analysis section. The yield of complexes, depending on protein concentration is shown in Fig. 3 in the range of A3G concentrations between 2 nM and 100 nM. The plot clearly demonstrates the dependence of yield of complexes on A3G concentration. The number of complexes increased rapidly and then plateaued, when the A3G concentration was greater than 16 nM.

### Deaminase Activity of A3G

**Figure 4:**
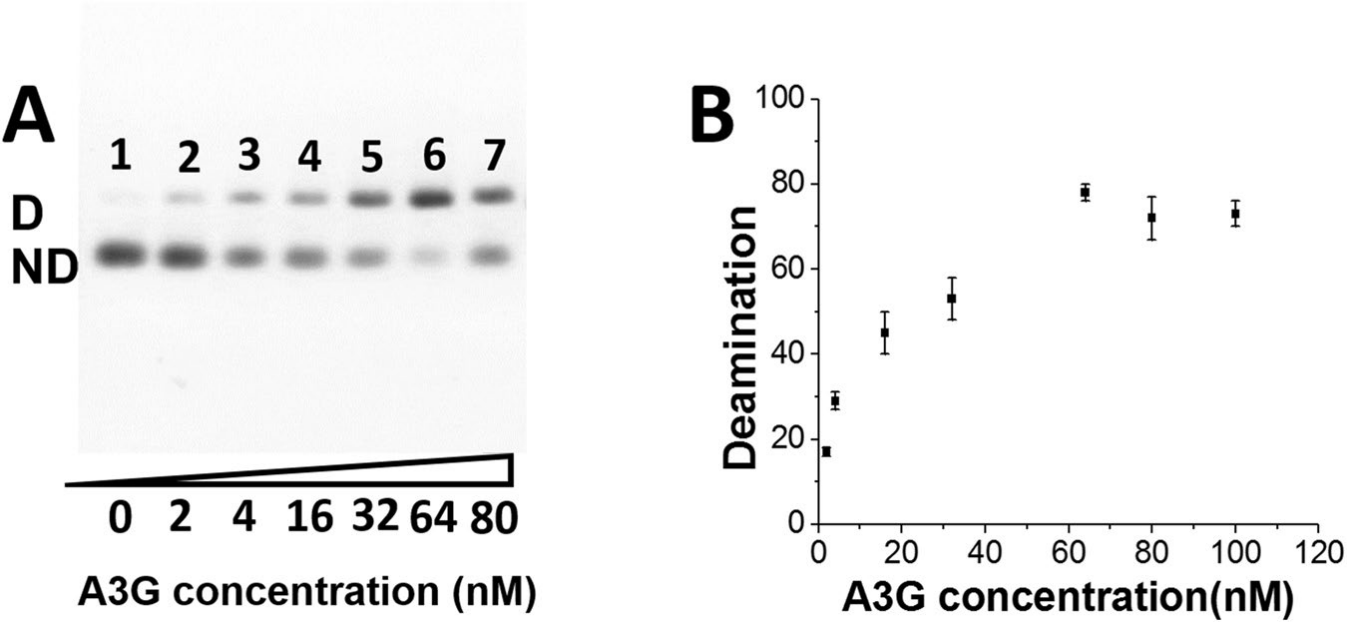
Agarose gel shows PCR products after digestion by SmaI enzyme. (**A**) Deaminase activity of A3G. Lane 1 shows 35 bp control after digestion of 70 bp PCR product without prior deamination; lanes 2 to 7 show the increase of deamination depending on A3G concentration (2 nM up to 80 nM). (**B**) The dependence of deaminase activity of A3G- ssDNA complexes on A3G concentration.

The deaminase activity of A3G was calculated using PCR deaminase assays, as described in the Material and Methods section. After A3G was mixed with 70 nt ssDNA, followed by PCR, two different PCR products were generated, which are schematically illustrated in Supporting Information Section Fig. S3A,B. The agarose gel in Fig. 4A shows the intensities for bands D and ND, depending on the efficiency of deamination by A3G. The band in lane 1, corresponding to a 35 bp fragment, serves as a control, which corresponds to complete digestion of 70 bp dsDNA by SmaI in the absence of A3G. Lanes 2–7 demonstrate that the intensity of band D increases, which correlates with an increase of A3G concentration in the deamination reaction with ssDNA. The agarose gel presented in Fig. 4A shows example for the analysis of PCR deaminase assay for A3G between 2 nM and 80 nM in the presence of 4 nM of ssDNA. All controls are shown in Supporting Information Section Fig. S4.

To quantify the deamination activity, we measured the intensity of bands deaminated (D) and non-deaminated (ND) and calculated the percentage of D to the sum of D + ND bands. The data are shown in Fig. 4B, where deaminase activity of A3G is plotted against the A3G concentration in the complex. The plot demonstrates a gradual increase in A3G deaminase activity in a range of A3G concentrations between 2 nM and 32 nM, and then reaches a plateau between 60 nM and 100 nM A3G.

## Discussion

In this paper, we identified the individual oligomeric states of wild type human A3G in complexes with ssDNA and estimated their deaminase activity. To achieve this goal, we used AFM for direct visualization of A3G-ssDNA complexes to determine the stoichiometry and the yield of complexes at different A3G concentrations. In parallel, we used PCR deaminase assay to estimate their deaminase activity. Importantly, we started with the lowest A3G concentration (2 nM), when A3G exists mostly as monomers in solution and kept the ssDNA template concentration at 4 nM for all reactions. Table 1 summarizes the data obtained for the total yield and total deaminase activity of A3G-DNA complexes depending on A3G concentration. At each A3G concentration, the mean values were calculated based on at least three independent experiments, obtained in parallel for the yield and deaminase activity of the complexes. The error was calculated as deviations from the mean values.

**Table 1:** Total yield and total deaminase activity of A3G-ssDNA complexes.

A3G concentration (nM)	Total yield of complexes (%)	Total deaminase activity (%)
2	24 ± 2	17 ± 1
4	40 ± 1	29 ± 2
16	69 ± 4	45 ± 5
32	74 ± 2	53 ± 5
64	78 ± 4	78 ± 2
80	70 ± 2	72 ± 5
100	70 ± 3	73 ± 3

Table 1 summarizes the mean values for the total yield and total deaminase activity of A3G complexes at different A3G concentrations. Data for the mean values were collected from at least three independent experiments. The errors show calculation as deviations from the mean values.

As shown in Table 1, we observed an increase in both the deaminase activity and yield of complexes when the concentration of A3G increased. Therefore, to compare the deaminase activity of A3G at a given A3G concentration, we must take into account the yield of the complexes at each concentration. To do that, we introduced the relative deaminase activity, which we determined as the ratio of total deaminase activity to the total yield of the complexes at each concentration of A3G. However, Fig. 2A–C, clearly shows the changes in the stoichiometry of A3G depending on its concentration. Indeed, at low A3G concentrations, between 2 nM and 4 nM, A3G exists in solution mostly as a monomer. Therefore, the relative deaminase activity of A3G monomer, as obtained from the ratio of total deaminase activity to the total yield of the complex (see Table 1), corresponds to 0.7 (at 2 nM: 17/24 and at 4 nM: 29/40). This result demonstrates, that monomer of wild type hA3G is catalytically active which fully support the data, obtained for deaminase activity of different mutants of monomeric A3G^13,18^.

At a concentration 16 nM, of A3G the maximum in the histogram (see Fig. 2B) shows mostly dimers of A3G in complexes with ssDNA, although the small admixture of monomers and trimers can be seen. The ratio of total deaminase activity to the total yield of such complexes results in 0.7 (16 nM: 45/69) for relative deaminase activity. The fact that the relative deaminase activity of A3G remains unchanged indicates that monomers and dimers of A3G have similar deaminase activity.

At increased concentrations of A3G in solution (32 nM), larger oligomers appeared in the A3G-DNA complexes. Figure 2C demonstrates the histogram for A3G stoichiometry at 32 nM, where A3G dimers (78%) coexist with tetramers (22%). After determining the relative deaminase activity for the dimer, calculated above as 0.7, we were able to quantify the relative deaminase activity for A3G tetramer. Table 1 shows that, at 32 nM A3G, the estimated total yield for all complexes was 74%. Therefore, between all complexes, 57% account for the dimers, and only 16% account for tetramers. The total deaminase activity of A3G at a concentration of 32 nM (see Table 1) was 53%. Based on the yield of the dimers at 57%, we estimated the total activity for the dimers at 32 nM to be 40% (0.7 × 57%). Subtracting the total activity for dimers from the total activity of all complexes at 32 nM (53–40%), we calculated 13% for the total deaminase activity of the tetramers. Therefore, relative deaminase activity of tetramers was equal to 0.8 (13%/16%).

At greater A3G concentrations (64 nM, 80 nM and 100 nM), however, A3G forms a mixture of different oligomers, which complicate the estimation of relative deaminase activity for individual A3G oligomers. Therefore, in this case, we estimated the relative deaminase activity for the mixture of A3G oligomers, as the ratio of the total deaminase activity to the total yield of the complexes, which resulted in the value ~1.0.

Table 2 summarizes the results for the calculated relative deaminase activity along with different oligomeric states of A3G in the complex. As it is seen from Fig. 2A–C, we assigned the oligomeric state at each concentration of A3G based on the maximum in Gaussian distribution. It is important that we started with the lowest A3G concentration, which results in a narrow distribution with the maximum correspond to A3G monomer. The raise of A3G concentration leads to the shift in the maximum and broadening in the histograms, which indicate on some heterogeneity of A3G oligomers, especially at higher A3G concentrations. However, the relative deaminase activity of oligomeric states (see Table 2) remains close to each other, which indicates that heterogeneity, does not affect the data. Indeed, if some of the oligomeric states have substantially larger or lower deaminase activity their contribution would change the outcome for the relative deaminase activity of A3G oligomers.

**Table 2:** The stoichiometry and calculated relative deaminase activity of A3G in the complex.

Stoichiometry of A3G	Relative deaminase activity
Monomer	0.7 ± 0.1
Dimer	0.7 ± 0.1
Tetramers	0.8 ± 0.1
Mixtures of oligomers	1.0 ± 0.1

For monomers, dimers and the mixture of A3G oligomers the relative deaminase activity was calculated as the ratio of total deaminase activity to the total yield of the complex. For the tetramers, the relative deaminase activity was calculated from the mixture of dimers (78%) and tetramers (22%) of A3G in the complex, as described in the text.

It is interesting that under 32 nM A3G concentration, A3G tends to form 2-mers and 4-mers, but at 80 nM A3G concentration, A3G tends to form 3-mers and 6-mers. It may suggest that the pathway for the assembly of higher order oligomers depends on A3G concentration in solution. Indeed, when the 2-mers of A3G presents most species in solution, there is more possibility to form 4-mers through dimer –dimer interactions. Similar pattern takes place when the majority of A3G are trimers. The trimer- trimer interactions are getting more prevalent, resulting in formation of 6-mers of A3G.

Although our results are fully in line with the data obtained in^18^, where authors suggested that A3G enzymatic activity is independent of multimerization, they are different from the data presented in^15^. One of the reasons for this discrepancy could be that the conclusion on the low deamination activity of oligomers in^15^ was made based on the comparative studies of the wild type A3G and its oligomerization deficient mutant. However, some mutants of A3G may have an elevated enzymatic activity as it is discussed in^22^. Our experiments were performed with wild type A3G and the same ssDNA substrates, also, deamination and oligomerization were measured in parallel experiments.

Based on the data obtained we propose the following model for A3G interaction and deamination. We recently build a computational model for the full sized A3G supported by high-resolution time-lapse AFM results^20^. According to this model, A3G has a highly dynamic structure in solution, changing from an extended, a dumbbell structure, to a compact, globular one. In the dumbbell structure of A3G, the NTD and CTD domains are visibly separated from each another connecting by a flexible linker with 8 residues (196–203). Based on these data, we speculate that such a structure allows CTD domain to efficiently search for the deamination spot on ssDNA substrate, while NTD domain remains strongly attached to the ssDNA substrate^20^. Morse *et al*.^15^ suggests that oligomerization of A3G occurs via the interactions between NTD domains. Therefore, based on such a model we hypothesize that one of the CTD domains in A3G oligomer may have an ability to search and deaminate the hot spot on ssDNA. Such a model could explain a similar deamination activity of monomer and oligomers of A3G.

In summary, we performed direct experiments to determine the deaminase activity of different oligomeric states of A3G and to the answer the question of whether the deaminase activity of A3G depends on protein stoichiometry. Our results clearly demonstrate that the wild type human A3G monomer is catalytically active, which overcomes controversy regarding the activity of the monomer. Moreover, it also shows that the dimers and tetramers of A3G have the catalytic activity similar to the one for the monomer, which suggests that oligomerization does not affect A3G deaminase activity, but at the same time may play an important role in deamination-independent mechanism.

## Methods

### DNA Hybrid Substrate Preparation

The hybrid DNA substrate was prepared as described in^11,20^. The schematics for assembly of hybrid DNA is presented in Fig. S1 of the Supporting Information Section. First, 89-nucleotide (nt) ssDNA and 23 nt ssDNA were synthesized by IDT (Integrated DNA Technology, Coralville, IA). Second, phosphorylated 23 nt was annealed with 89 nt ssDNA at a 1:1 ratio to produce a duplex with three sticky ends. Third, the annealed product was ligated overnight at 4 °C with a previously gel-purified 356 base pairs (bp) double – stranded DNA (dsDNA) fragment, containing three sticky ends. Finally, the ligated hybrid DNA containing 69 nt ssDNA and 376 bp ds DNA part was purified from 1.5% agarose gel as describe in^20,23^, and stored at −20 °C. The AFM image of hybrid ssDNA substrate is shown in Supplement Information Section as Fig. S2.

### Preparation of Complexes of A3G with Hybrid DNA and 70 nt ssDNA

The full length human APOBEG3G was purified as described by Li *et al*.^24^. Two ssDNA substrates were used in this study. First, a hybrid ssDNA substrate, assembled as described above, containing 69 nt ssDNA for A3G binding and 376 bp dsDNA, used as a tag^20^ for clear identification of A3G-DNA complexes with AFM. Second, synthesized by IDT (Integrated DNA Technology, Coralville, IA) 70 nt ssDNA, containing one hot spot for deamination by A3G was used to estimate deaminase activity of A3G-ssDNA complexes. To avoid possible errors with A3G dilutions, the A3G-hybrid DNA complexes and A3G-70 nt ssDNA complexes were prepared in parallel in binding buffer, containing 50 mM HEPES (pH 7.5), 100 mM NaCl, 5 mM MgCl_2,_ 1 mM DTT and incubated at 37 °C for 30 min. The concentration of DNA in the reactions was kept constant at 4 nM and A3G concentration varied from 2 nM up to 100 nM.

### Sample Preparation for AFM Imaging

AFM experiments were performed with the use of functionalized 1-(3-aminopropyl)silatrane (APS)-mica as described^11,12,23^. Briefly, a 5 μl sample was deposited on APS-mica for 2 min, rinsed with deionized water and dried with Argon gas. Images were acquired in tapping mode in air using the Multimode Nanoscope IV system (Bruker-Nano; Santa Barbara, CA). Silicon probes with 42 N/m at resonance frequencies between 310–340 Hz were used.

### Data Analysis

AFM images were used to estimate the yield of complexes and stoichiometry of the protein in the complex with hybrid DNA as described in details in^25^. For each protein concentration, the volume and yield of the complexes were obtained using FemtoScan Online software (Advance Technologies Center, Moscow, Russia). The yield of the complexes, in percentages was calculated as the ratio of A3G-DNA complexes to the total number of DNA molecules, including complexes with A3G, on each AFM image. The protein volume was calculated by measuring the height and width of protein, using the cross-section feature from FemtoScan, as used in previous studies^25^. From the measured height and width of the protein, the protein volume was determined by an equation described by Henderson *et al*.^26^. The data for protein volumes were assembled as histograms using Origin 6.0 (OriginLab, Northampton, MA) and converted into the molecular mass of protein using a conversion coefficient 1.3 as calculated by Shlyakhtenko *et al*.^21^.

### Deaminase Assay by PCR

To estimate the deaminase activity of A3G we used PCR deaminase assay, similar to the assay, described in^10^. The following 70 nt sequence with a hot spot for deamination, located in the center (in bold) was used:

5′GCGCAGTGGTACGCGTATTTGAGAAGAGATAA**CCCGGG**ATGAATGAAAAAGAAGAGCCGCGTTG CTGTCG-3′.

For the deamination reaction, 200 µL of 70 nt ssDNA at 4 nM concentration was mixed with varying amounts of A3G (from 2 nM to 100 nM) in binding buffer, containing 50 mM HEPES, 150 mM NaCl, 5 mM MgCl_2_, 1 mM DTT and incubated for 30 minutes at 37 °C. Then, 12.5 µL of reaction mixture was used for PCR amplification, with the following program: 1 cycle at 94 °C for 2 min, followed by 33 cycles of annealing at 53 °C for 30 sec, denaturing at 94 °C for 30 sec and elongation at 72 °C for 70 sec. The forward and reverse primers were the follow: 5′GCG CAG TGG TAC GCG3′ 15nt (forward), 5′-CGA CAG CAA CGC GGC-3′ 15 nt (reverse). After PCR reaction, QIAquick Gel Extraction Kit (QIAGEN Inc., MD 20874, USA) was used to purify dsDNA. The purified dsDNA, digested by SmaI restriction enzyme (New England Biolab, MA 01938, USA) at room temperature for 1 h, was loaded on 2% agarose gel. The details of the assay are schematically presented in the Supporting Information sections, Fig. S2A,B. Two different 70 bp dsDNA products were amplified after PCR reaction. First, if A3G did not deaminate ssDNA, 70 bp dsDNA will preserve CCCGGG site for the restriction by enzyme SmaI. In this case, after restriction, only one band was expected on the gel, which was marked as non-deaminated band (ND) in Fig. S3A. Second, if A3G deaminated ssDNA, then C, in the middle of the sequence, will change for U and after PCR reaction, the product should not have site for the SmaI restriction enzyme (*i*.*e*., CCTGGG). Figure S3B shows step-by-step reactions in case of A3G deamination took place. In this case, two bands will show on the gel: non-deaminated (ND) and deaminated (D) respectively, and their intensity varied depending on the concentration of A3G. The agarose gel used for estimation of deaminase activity is shown in Support Information section Fig. S4.

### Electrophoretic Mobility Shift Assay (EMSA)

The 2% agarose gel was visualized by UV light and analyzed by Image J software (NIH). The percentage of A3G deaminase activity was determined as the ratio of intensity for deaminated band D to the total intensity for deaminated and non-deaminated bands D/(D + ND).

## Electronic supplementary material

The Enzymatic Activity of APOBE3G Multimers.
