# Supplementary material for: The Enzymatic Activity of APOBE3G Multimers
Source: Sci Rep. 2018 Dec 18;8:17953. doi: 10.1038/s41598-018-36372-6 (PMC6298963; doi:10.1038/s41598-018-36372-6)
Supplement: Supplementary file 1 — The Enzymatic Activity of APOBE3G Multimers. [file 41598_2018_36372_MOESM1_ESM.pdf]

Supporting Information

The Enzymatic Activity of APOBE3G Multimers.

Yangang Pan[1], Karen Zagorski[1], Luda S. Shlyakhtenko[1], and Yuri L. Lyubchenko[1*].*

Assembly of hybrid DNA substrate

1) 89nt synthetic oligos

5' TAC GTG TAG GAA TTA TAT TAA AGA GAA AGT GAA ACC CAA AGA ATG AAA AAG AAG ATG TTA GAA TTG TTA GCG GTA TCA GCT CAC TCA TA

2) Synthetic 23nt ssDNA for annealing with 20 nt ssDNA.

5' GCT TAT GAG TGA GCT GAT ACC GC

3) Annealing of 23 nt ssDNA (2) with 89 nt oligos (1) to create partial 20 bp dsDNA region (shown as blue thick lines) with sticky end (shown with dotted line) and 69 nt DNA tail (thin blue line).

4) Ligation of annealed 20 bp dsDNA containing 69 nt DNA and sticky ends with 356 bp dsDNA fragment, containing complementary sticky end (shown as red thick lines and dotted line), to get 379 bp dsDNA tag with 69 nt DNA tail.

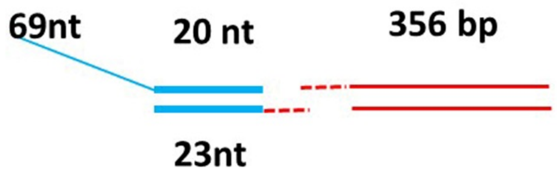

Figure S1. Assembly of hybrid DNA substrate. Four steps present the assembly of the hybrid DNA substrate**.** Total hybrid DNA has 379bp dsDNA and 69nt ssDNA.

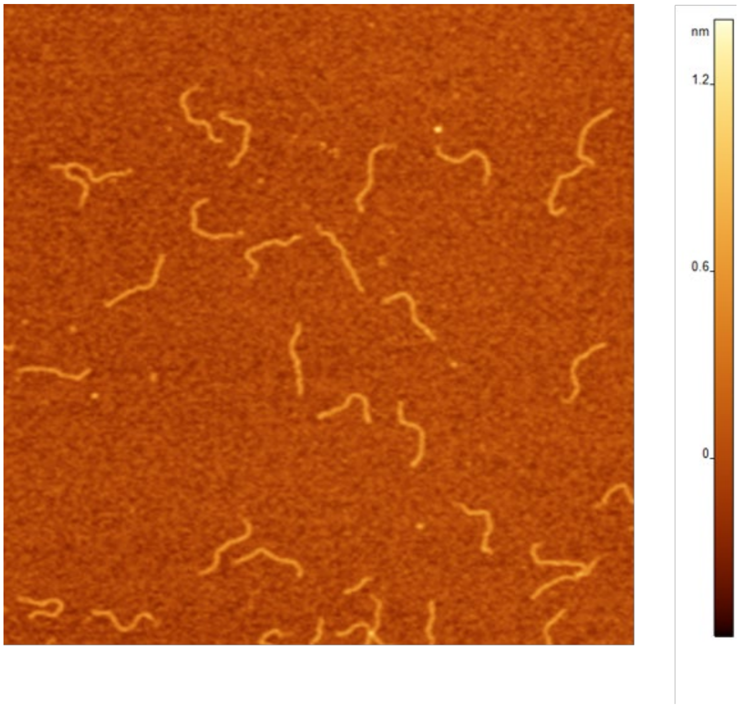

Figure S2. The AFM image of hybrid DNA deposited on APS mica without A3G. The image size is 1 micron.

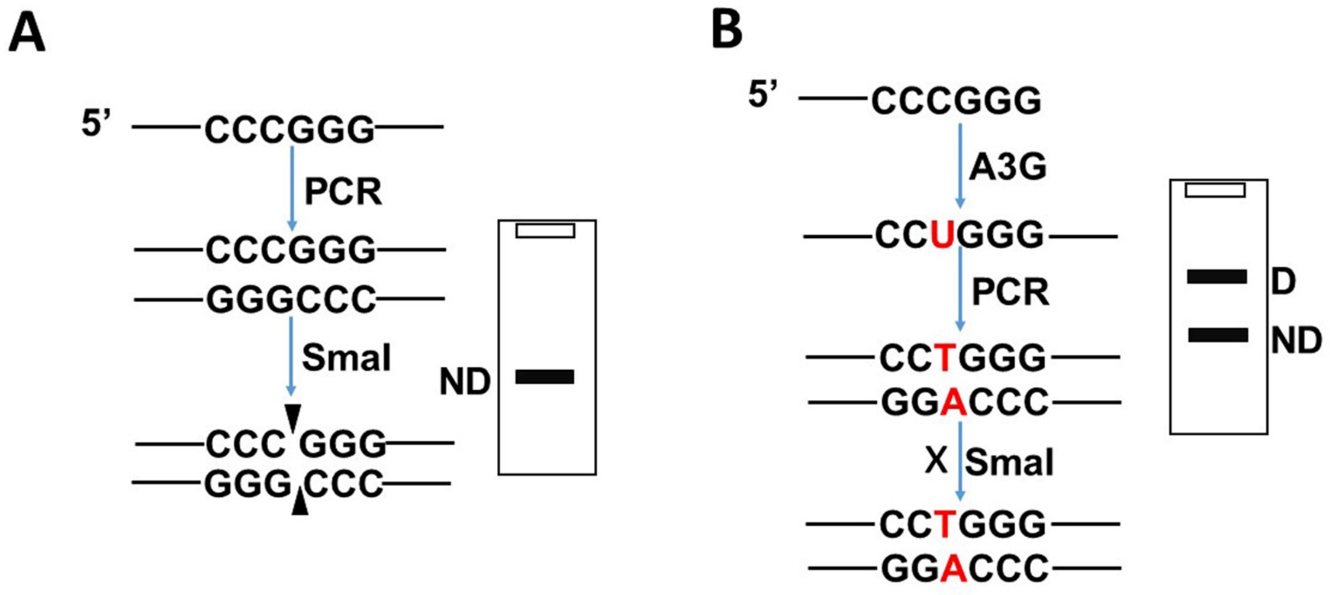

(5' GCGCAGTGGTACGCGTATTTGAGAAGAGATAA**CCCGGG**ATGAATGAAAAAGAAGAGCCGCGTTG CTGTCG- 3)'

Figure S3. Schematics of deamination assay. A-complete DNA cleavage by SmaI restriction enzyme without deamination reaction shows one non-deaminated band ND. B- partial deamination reaction shows deamination band (D) and non-deaminated band (ND).

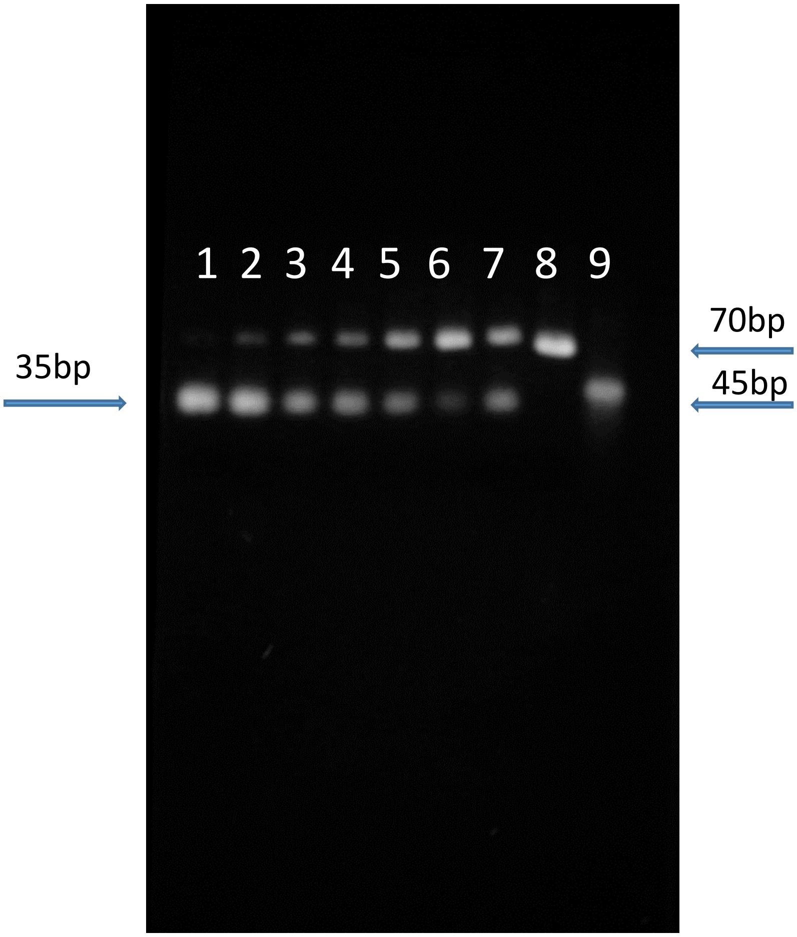

Figure S4. Agarose gel used for the analysis of PCR deaminase assay. Lane 1- 35 bp dsDNA fragment resulted in digestion of 70 bp dsDNA fragment by SmaI restriction enzyme in the absence of A3G (negative control). Lanes 2-7 – the deamination reaction between A3G (starting from 2nM up to 80nM) and 4nM of ssDNA. Lane 8- 70 bp dsDNA marker. Lane 9- 45 bp dsDNA marker.

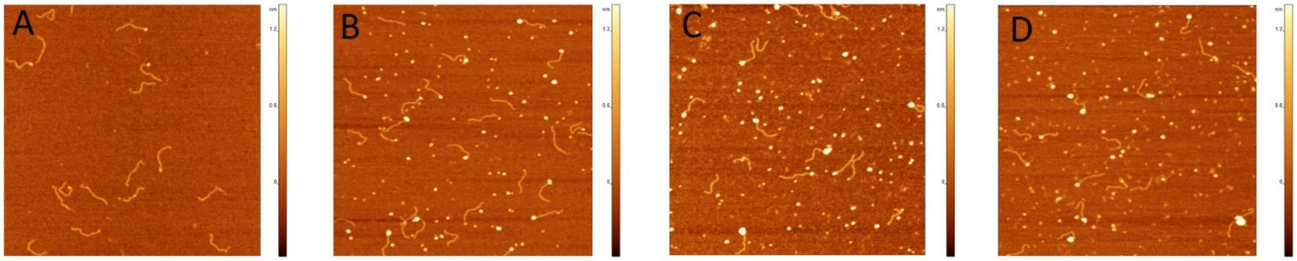

Figure S5. The AFM images of A3G with 4 nM ssDNA. Concentrations of A3G: 2nM (A), 32nM (B), 80nM (C) and 100nM (D). The image size is 1 micron.
